# *Mycobacterium tuberculosis* Rho Is an NTPase with Distinct Kinetic Properties and a Novel RNA-Binding Subdomain

**DOI:** 10.1371/journal.pone.0107474

**Published:** 2014-09-17

**Authors:** Anirban Mitra, Rachel Misquitta, Valakunja Nagaraja

**Affiliations:** 1 Department of Microbiology and Cell Biology, Indian Institute of Science, Bangalore, India; 2 Jawaharlal Nehru Centre for Advanced Scientific Research, Bangalore, India; Indian Institute of Science, India

## Abstract

Two mechanisms – factor independent and dependent termination – ensure the completion of RNA synthesis in eubacteria. Factor-dependent mechanism relies on the Rho protein to terminate transcription by interacting with RNA polymerase. Although well studied in *Escherichia coli*, the properties of the Rho homologs from most bacteria are not known. The *rho* gene is unusually large in genus *Mycobacterium* and other members of actinobacteria, having ∼150 additional residues towards the amino terminal end. We describe the distinct properties of Rho from *Mycobacterium tuberculosis*. It is an NTPase with a preference for purine nucleoside triphosphates with kinetic properties different from *E. coli* homolog and an ability to use various RNA substrates. The N-terminal subdomain of MtbRho can bind to RNA by itself, and appears to contribute to the interaction of the termination factor with RNAs. Furthermore, the interaction with RNA induces changes in conformation and oligomerization of MtbRho.

## Introduction

Termination of transcription of bacterial RNA polymerase (RNAP) is achieved either by intrinsic terminators or protein factor Rho. For factor-mediated termination, Rho binds to the nascent transcript emerging from the ternary elongation complex, translocates along the RNA by ATP-powered steps and finally enforces dissociation of the complex [1], [2], [3], [4]. The RNA-binding and ATPase properties of the prototype Rho factor from *Escherichia coli* have been studied extensively [5], [6], [7]. Briefly, *E. coli* Rho (EcRho) is functionally a homohexameric molecule [8] [9] that preferentially binds to an unstructured, C-rich RNA. This interaction induces transition from an ‘open’ ring to ‘close’ ring state [10]. The closed ring is proficient in ATP hydrolysis and translocation along RNA. Once it catches up with the transcribing or paused RNAP, the interaction triggers termination, dissociation of RNAP from the template and release of the transcript [11], [12], [13], [14]. Several studies on EcRho have unraveled the biochemical and structural basis for its preference for C-rich RNA [15], [16], [17], [18], [19], [20]. However, in spite of its key cellular role and its presence in a large number of diverse bacterial families [21], very few Rho homologs have been studied. Characterization of the properties and understanding Rho-mediated termination is of paramount importance in organisms such as *Mycobacterium tuberculosis* (*Mtb*) [22] which is the causative agent of the number-one killer disease worldwide.

In addition to *Mtb*, the genus *Mycobacterium* includes some of the well-known Actinobacteria, such as the well-studied model organism *Mycobacterium smegmatis, Mycobacterium abscessus*, *Mycobacterium leprae*, and a large number of *Streptomyces* species. In recent years, studies on transcription initiation, elongation and termination have been carried out in *Mtb* and other members. These studies, though not as exhaustive as in *E. coli*, have revealed considerable differences from the *E. coli* paradigm. For example, the *Mtb* and other mycobacterial genomes code for a larger number of sigma factors [23] as compared to *E.coli* and also for the several transcription factors unique to mycobacteria [24], [25]. Several promoters and the mechanism of gene expression regulation have been studied [26], [27], [28], [29], [30]. Absence of AT-rich UP elements and GC-rich sequences in discriminator sequences in the promoters contribute to the differences in promoter-polymerase interaction and its regulation [31], [32], [33], [34], [35]. Additionally, attempts have been made to elucidate features of RNAP from *Mycobacterium* species [36], [37], [38] and the transcription elongation rates also appear to vary between different RNAPs [39]. Furthermore, the scarcity of canonical intrinsic terminators and an abundance of non-canonical intrinsic terminators across mycobacteria also suggest differences in the transcription termination machinery [40], [41].

Given the dissimilarities in various steps of transcription between mycobacteria and *E. coli*, it is likely that mycobacterial Rho homologs have also evolved to function differently and optimally for their specific cellular context. Notably, sequence analysis showed that the Rho homologs in *Mycobacterium* species and other actinobacteria are larger than EcRho mainly due to an ‘extra-stretch’ of ∼150–200 residues in their RNA-binding domains [5], [7], [21], [22], [42], [43]. In this manuscript, we present results demonstrating that purified *M. tuberculosis* Rho (MtbRho) can hydrolyse purine nucleoside triphosphates(NTP) – ATP and GTP-in presence of mycobacterial RNA. The extended N-terminal region of MtbRho, having a distinct RNA-binding ‘subdomain’, can itself interact with RNA and may contribute to the overall interaction. The MtbRho-RNA interactions are stable and the interactions induce changes in the conformation and oligomerization status of the protein.

## Results

### MtbRho can hydrolyse NTP in presence of mycobacterial RNA

The presence of Rho homologs in diverse bacterial lineages (Figure S1A) indicated the functional importance of factor-dependent termination in bacterial gene expression. Analysis of Rho sequences showed that while the C-terminal half is well-conserved, the N-terminal half is more variable. Notably, the Rho homologs from actinobacteria form a distinct branch and the N-terminal halves of actinobacterial rho proteins contain an ‘extra-stretch/subdomain’ of 150–200 residues. The sequence composition of this fragment includes a large number of Arg, Asp, Asn and Gly residues but very few hydrophobic and aromatic amino acids [5], [7], [22]. Although no function can be conclusively predicted from the sequence analysis, its role in interacting with RNA is postulated from the presence of a large number of basic amino acid residues [7], [42] (Figure S1B). To understand the properties of the Rho protein from *M. tuberculosis*, we expressed recombinant MtbRho in BL21(DE3) cells and purified it to homogeneity. MtbRho has a monomeric size of 65 kDa (Figure S2), as estimated from sequence analysis and also shown by mass spectrometry. However, the protein showed anomalous migration at ∼80 kDa on SDS-PAGE [22], probably due to the presence of clusters of polar residues in the subdomain.

**Figure 1:**
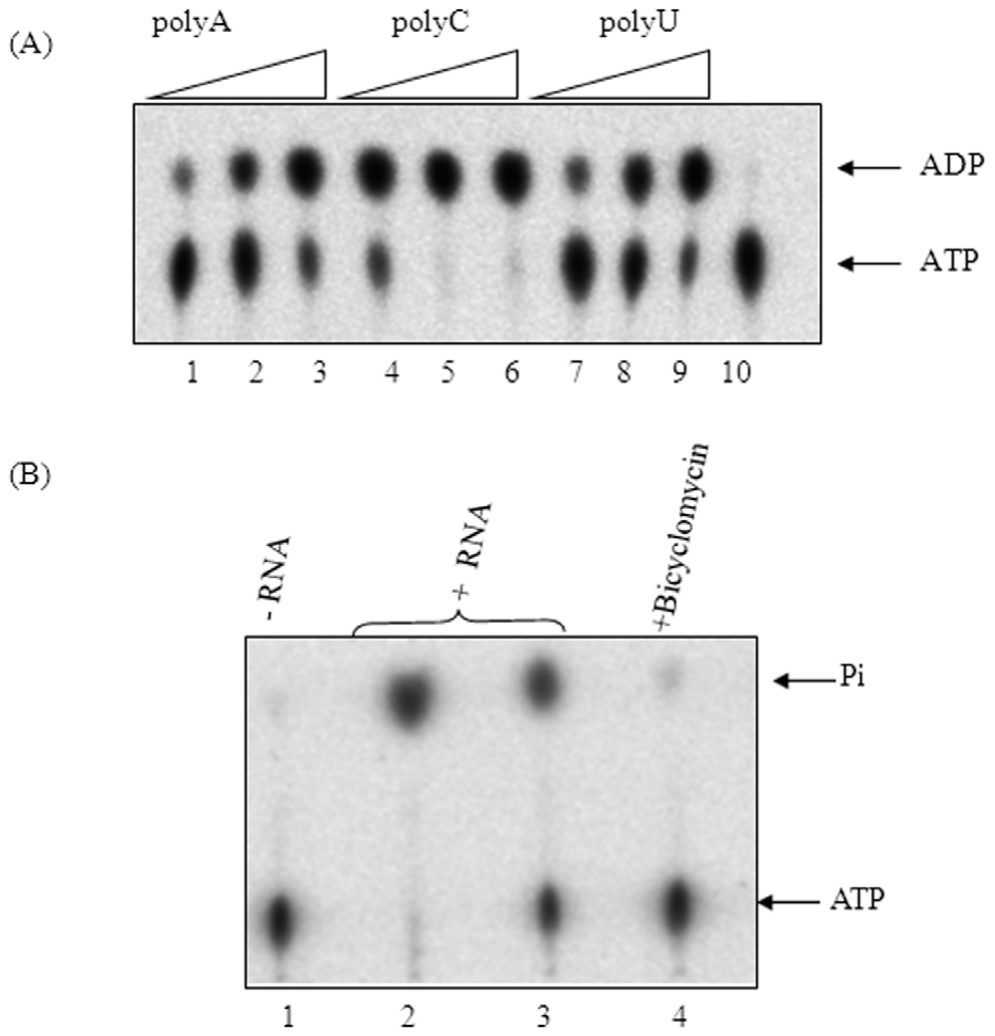
ATPase activity of MtbRho. (A) MtbRho hydrolyses ATP in presence of increasing concentrations of homopolymeric RNA – polyA (lanes1–3), polyC (lanes4–6) and polyU (lanes 7–9). No hydrolysis was observed in absence of RNA (lane 10). (B) MtbRho hydrolyzes ATP in presence of mycobacterial RNA. No hydrolysis was observed in absence of RNA (lane 1); 2 and 1 µg of *M. smegmatis* RNA stimulated ATP hydrolysis (lanes 2,3); the reaction is inhibited by Bicyclomycin (lane 4). ATPase assay was carried out as described in Methods. [1 mM unlabeled ATP was used as substrate, along with 100 nCi of α-^32^P-ATP (Panel A) or 100 nCi of γ-^32^P-ATP(Panel B), as tracer. Hydrolysis resulted in formation of α-^32^P-ADP (Panel A) or ^32^Pi (Panel B) which were visualized using phosphorimager].

**Figure 2:**
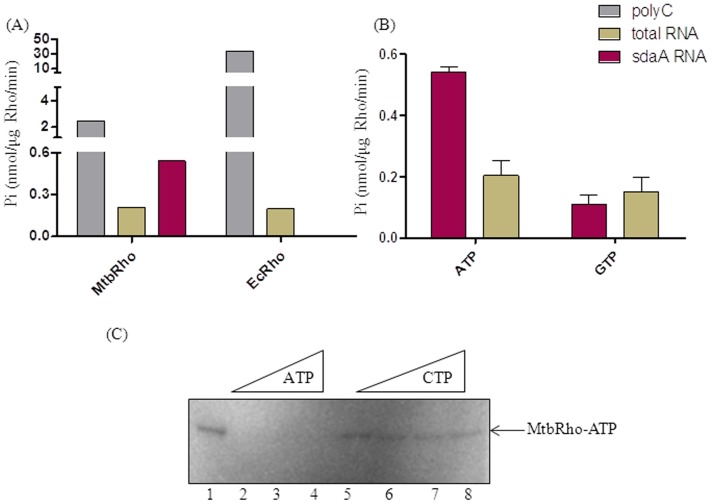
RNA preference by MtbRho. (A) Rates of ATPase activity of MtbRho and EcRho in presence of different RNAs. MtbRho is a weaker ATPase when polyC)is used(grey bars). But, in presence of mycobacterial RNA (brown bars), they have comparable rates of hydrolysis, while only MtbRho can hydrolyse ATP in presence of *sdaA* RNA(red bars). (B) Differential NTP hydrolysis by MtbRho. MtbRho can hydrolyze ATP and GTP in presence of the mycobacterial total RNA and *sdaA* RNA. Colorimetric assay was carried out as described in Methods (C) Differential ability of MtbRho to bind ATP and CTP. γ-^32^P-ATP alone can be UV-crosslinked to MtbRho (lane 1), and visualized on 8% SDS-PAGE. Addition of unlabeled ATP to the reaction competes out the γ-^32^P-ATP (lanes 2–4), but unlabeled CTP fails to do so (lanes 5–8). The reaction conditions were similar to ATPase assay, but without the addition of RNA.

In the several steps involved in Rho-mediated transcription termination, the first step of Rho's action is its binding to the rut (rho utilization) site [7]. EcRho is known to have a preference for C-rich, unstructured RNA for initial Rho binding [16] and polycytidylic acid (polyC) has been used to study Rho activity *in vitro* [15], [19], [44], [45], [46], [47], [48]. The residues of EcRho that have been implicated in binding to a C-rich sequence (5′-CC YC-3′) [18] the motifs involved in RNA-dependent ATPase activity are similar in MtbRho. The ATPase activity of MtbRho in presence of synthetic homopolymeric polyC, polyA and polyU is shown in Figure 1A. While polyC is, not unexpectedly, the best substrate, polyA and polyU also stimulate the hydrolysis of ATP(Figure 1A). Homopolymeric polyC, polyA and polyU are, however, not natural substrates of MtbRho. *In vivo*, MtbRho would function in presence of various mycobacterial RNAs and it is likely that MtbRho has evolved a greater ability to interact with its natural substrates. To assess if MtbRho could use mycobacterial RNA as substrate for ATPase, cellular RNA from *M. smegmatis* mc^2^155 was used. The results presented in Figure 1B show that MtbRho can hydrolyse ATP in presence of mycobacterial RNAs. The ATPase activity was specific to MtbRho as it was inhibited by Bicyclomcycin. To study if a specific mycobacterial RNA molecule could be used as a substrate for ATPase activity, we used a RNA corresponding to the region downstream of the *sdaA* gene of *M. tuberculosis* genome. This RNA was chosen as *in silico* analysis revealed the absence of intrinsic terminator downstream of the *sdaA* gene [49] and hence it is likely target for Rho-dependent termination. The results presented in Figure 2A and B show that MtbRho can hydrolyse ATP in presence of *sdaA* RNA.

But, MtbRho is inherently a weaker ATPase [22]. The rate of ATP hydrolysis by MtbRho in presence of polyC was >10-fold less when compared to that of EcRho (Figure 2A). Also, as previous studies have shown, MtbRho exhibited a higher K_m_ (75.2 µM) for ATP than EcRho (10 µM) [22]. The slow rate of ATP hydrolysis indicates the intrinsically low activity of the enzyme. However, when mycobacterial cellular RNA was used, the ATPase rate of MtbRho became similar to previous observations where *E. coli* terminator RNAs had been used [22]. Remarkably, the rates of MtbRho and EcRho became comparable in presence of mycobacterial cellular RNA (Figure 2A). This indicated that MtbRho could be more proficient in using mycobacterial RNA as substrate than its *E. coli* homolog. The superior ability of MtbRho to utilize mycobacterial RNA was further evident when, in presence of *sdaA* RNA, a specific *Mtb* RNA, MtbRho hydrolysed ATP at a rate that is 2-fold higher than that reported in presence of the *E. coli* terminators [22], while EcRho was unable to use the *sdaA* RNA for ATPase activity (Figure 2A). Thus, although a weaker ATPase in presence of polyC, MtbRho seems to be more efficient in catalysis in presence of a specific RNA from *M. tuberculosis* that is likely to be its natural substrate (Figure 2A). Besides ATP, MtbRho hydrolysed GTP while RNA-dependent CTP and UTP hydrolysis was undetectable (Figure 2B) [22]. Thus, MtbRho can be considered an NTPase, with a substrate preference for ATP and GTP, when provided with its cognate RNAs. The ability to hydrolyze the various NTPs at different levels was mirrored by MtbRho's ability to bind the various NTPs with varying efficiency. When UV-radiation was used to crosslink γ-32P-ATP to MtbRho, in presence of unlabeled NTPs, ATP could compete out the crosslinking of γ-32P-ATP, but CTP could not (Figure 2C).

### N-terminal subdomain binds to RNA

**Figure 3:**
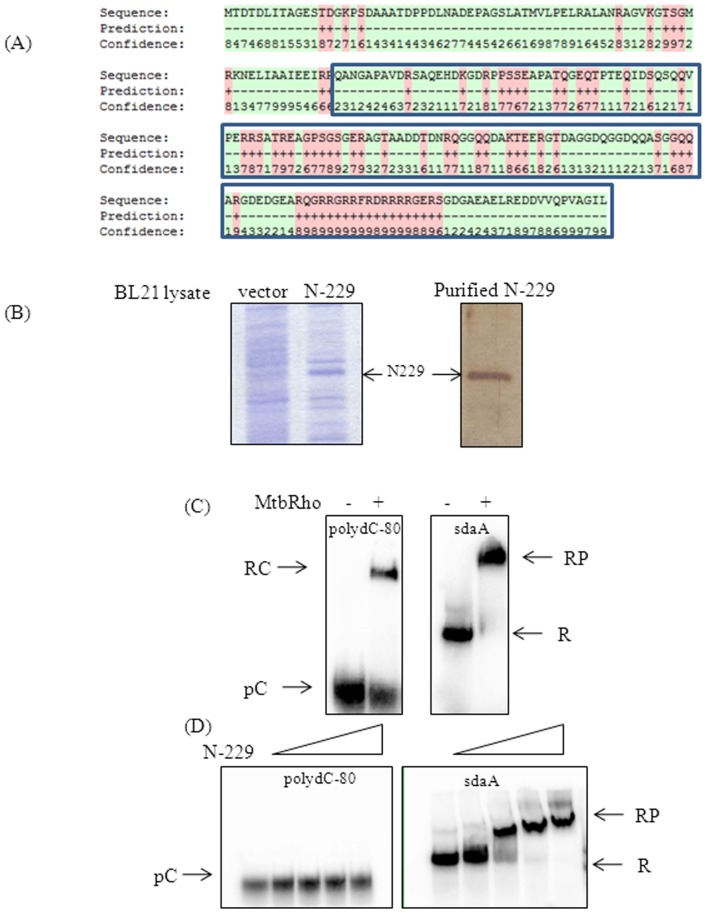
The subdomain of MtbRho contributes to RNA-binding. (A) Sequence of the N-229. The extra-stretch is shown within blue-boxes. The residues predicted to bind to RNA are labeled with ‘+’ and ‘red’. Values 0–9 indicate a gradient of confidence in prediction. The *in silico* analysis was carried out using the BindN web-server(50). (B) N-229 fragment was overexpressed from the pET20b clone in BL21 cells. Lysate of pET20b-N229 shows expression (N-229), as compared to control lysate (vector). N-229 was purified to homogenity, shown by silver staining. (C) MtbRho binding to RNA. MtbRho binds to both poly-dC80 and mycobacterial *sdaA* RNA. (D) N-229 binding to RNA. N-229 binds only to *sdaA* RNA. The RNA-protein complexes(RP, RC) were resolved from free RNA (R, pC) by 4% native PAGE.

All actinobacterial Rho homologs carry a 150–200 amino acid long subdomain in the N-terminal region, located immediately upstream of the canonical RNA-binding motifs, which has been implicated in assisting to bind RNA [7]. By sequence comparison, the MtbRho's subdomain spans the residues 76–230. To understand its role in the functionality of MtbRho, the N-terminal 229 residues (N-229), which includes the additional sequences was expressed and purified. Notably, although the N-229 lacks the canonical RNA-binding motifs of Rho, *in silico* analysis predicted that several patches of amino acids in this fragment may interact with a RNA substrate (Figure 3A) [50]. To confirm the interaction of N-229 with mycobacterial RNA, binding was monitored with a defined mycobacterial RNA molecule. The substrate RNA was *in vitro* transcribed ^32^P-labeled *sdaA* RNA. Predictably, full-length MtbRho bound to *sdaA* RNA (Figure 3C). Although N-229 lacks both the primary and secondary RNA-binding residues present in all Rho homologs, it could stably interact with the 351-nucleotide long *Mtb sdaA* RNA(Figure 3D). However, the binding pattern with 80-mer polydC-80 was significantly different. Although MtbRho would bind to poly-dC80 (Figure 3C), in contrast, N-229 showed very weak interaction with poly-dC80 (Figure 3D). The binding of the N-terminal fragment of MtbRho could be visualized only if the complex is stabilized by the addition of 20% glycerol to the gel matrix (Figure S3). Accordingly, in the MtbRho-dependent ATPase assays, the N-terminal fragment inhibited ATPase activity to a low extent (data not shown).

### Conformational changes in MtbRho associated with RNA binding

The presence of the mycobacterial RNAs conferred protection to MtbRho when probed with V8 protease (Endoproteinase Glu-C) (Figure S4A). Both cellular RNA, which contains several large RNA species and smaller tRNA (average size ∼100 nucleotides) protected MtbRho from cleavage by V8 protease. The RNA-induced protection is indicative of conformational changes in MtbRho upon RNA-binding rendering certain scissile sites inaccessible to the protease. The RNA-based protection also shows close interaction between the substrate RNA and MtbRho, which sterically hinders cleavage by V8 protease action. The addition of ATP along with RNA did not induce further differences in protease protection pattern and ATP, by itself, did not confer any protection. In order to investigate conformational changes induced in MtbRho by RNA, circular dichroism studies (CD) were carried out. There was a distinct alteration of secondary structures in presence of RNA (Figure S4B). In contrast, CD spectra of MtbRho in presence and absence of its substrate ATP was unchanged, indicating that ATP alone did not induce any significant conformational changes. Thus, both protease protection and CD studies, show RNA-induced conformational changes in the protein.

**Figure 4:**
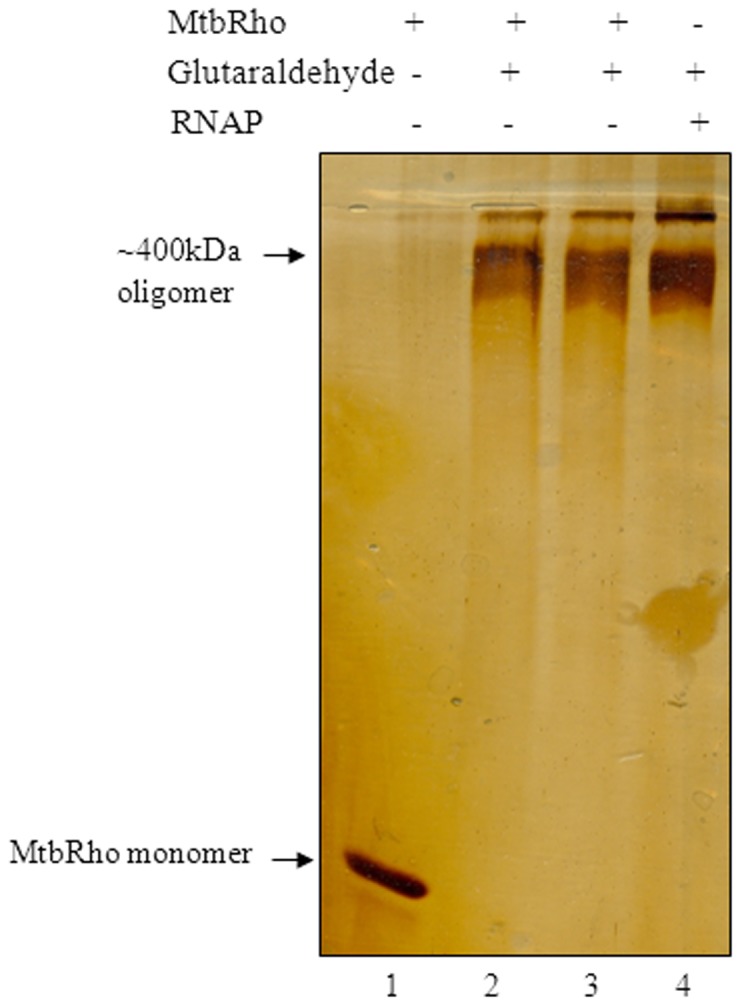
Oligomerization status of MtbRho. 2 µg MtbRho crosslinked by glutaraldehyde (0.05%) for 5 min and 10 min (lanes 2,3). Uncrosslinked MtbRho migrated as monomer (lane 1). RNAP was crosslinked and used as a marker of MW ∼400 kDa (lane 4). Reaction buffer was similar to that in ATPase assay, except that KCl replaced K-Glutamate. Products were resolved in gradient SDS-PAGE.

**Figure 5:**
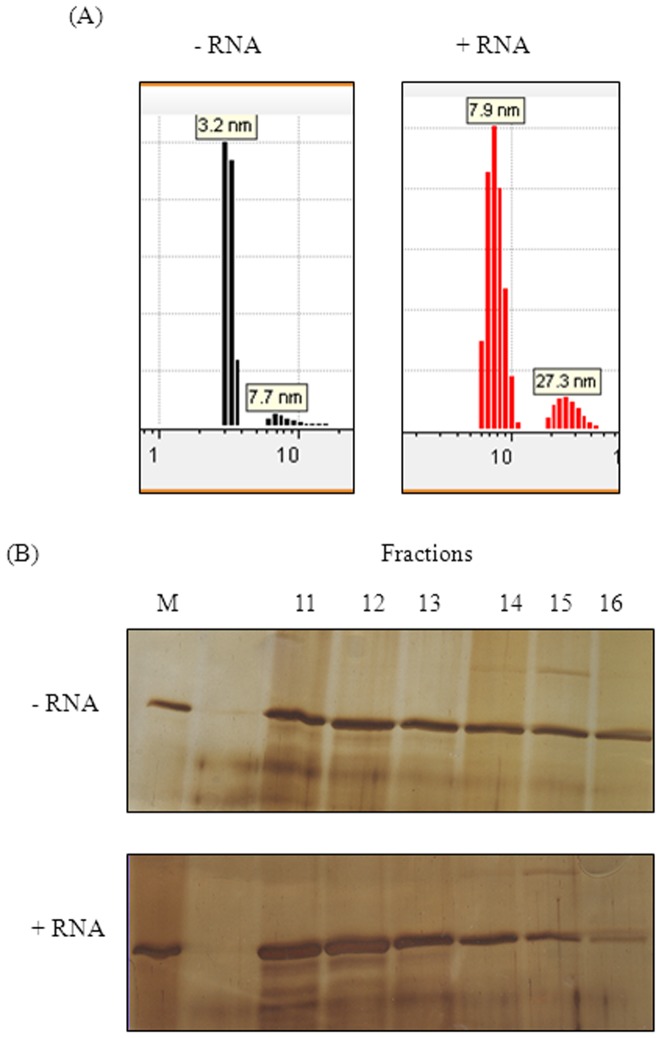
RNA induces hexameric form of MtbRho. (A) Dynamic Light Scattering profile of MtbRho. MtbRho was subjected to DLS alone (-RNA) or in presence of RNA (+RNA). A 10∶1 molar ratio of RNA: Rho was maintained. The histogram represents the intensity distribution of the MtbRho. The RH values of 3.2 and 7.7–7.9 nm corresponds to MtbRho monomer and hexamer respectively; 27.3 nm corresponds to protein aggregate. (B) Analysis of Gel filtration fractions. SDS-PAGE profile of fractions eluted from by Superdex S200 column, with MtbRho alone (-RNA), or after incubating with RNA and ATP (+RNA). MtbRho elutes between fractions 11–16 in both cases, but is shifted towards higher oligomers in presence of RNA.

Treating MtbRho with glutaraldehyde, a bifunctional crosslinking agent, showed that crosslinked MtbRho migrates with a mobility corresponding to a mass of ∼400 kDa. Since the MtbRho monomer is 65 kDa, the crosslinked product observed is likely to be hexameric (Figure 4). The functional state of all Rho homologues studied so far is hexameric in nature [8], [9], [10]. Dynamic light scattering (DLS) and analytical gel filtration studies further highlighted the flexible nature of the protein. The DLS results indicated that, in absence of substrate RNA, >90% of MtbRho existed as a monomer in solution (RH 3.2 nm) (Figure 5A). In contrast, addition of RNA shifted 87% of the molecules towards a RH value of 7.7 nm, which corresponds with a hexameric form. Thus, RNA induced MtbRho monomers to stably adopt the hexameric conformation (Figure 5A). Similarly, analytical gel filtration showed that MtbRho existed both as monomer as well as oligomers in solution. However, a distinct shift towards formation of oligomeric forms was seen in the presence of RNA (Figure 5B), indicating that MtbRho is a dynamic molecule and interaction with RNA promotes the formation of the functionally active hexamer.

### MtbRho can terminate transcription by EcRNAP, but Mtb rho cannot complement E. coli rho

**Figure 6:**
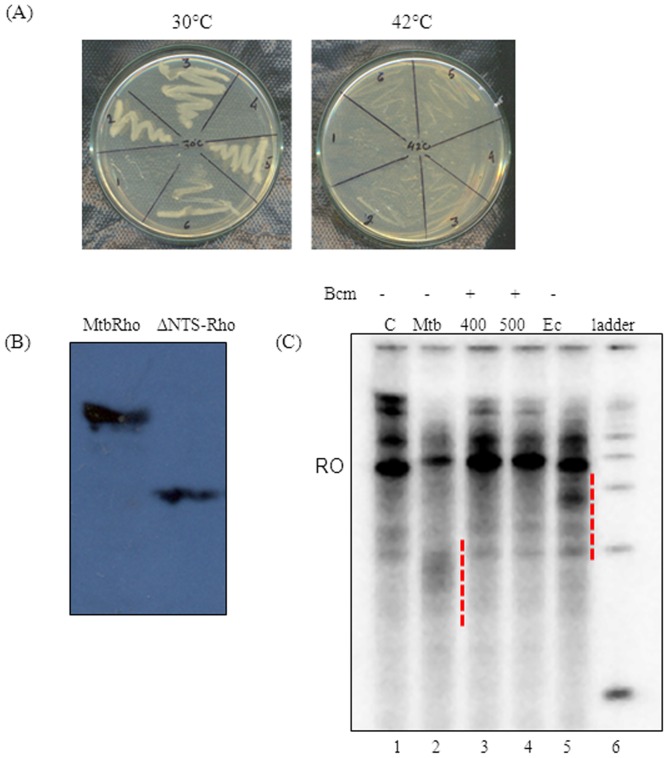
MtbRho is specific to *M. tuberculosis.* (A) Complementation of *E.coli* AMO14 strain attempted with Mtb *rho*. Transformants harbouring pTrc99C or pTrc99C-*Mtbrho* were grown to OD_600_ 0.6 at 30°C, streaked on IPTG- containing plates and incubated at permissive (30°C) or non-permissive (42°C) temperature. Untransformed cells(1,4); transformants with pTrc99C (2,5); transformants with pTrc99C-*Mtbrho*(3,6).(B) Expression of MtbRho and ΔNTS-MtbRho in *E.coli* AMO14 transformants when induced with 0.3 mM IPTG at permissible temperature. (C) MtbRho terminates transcription by *E. coli* RNAP *in vitro*. Run-off transcript (RO) was reduced and several smaller transcripts appeared when transcription was done in presence of MtbRho (Mtb; lane 2) compared to that in absence of MtbRho (C; lane 1). The effect was reversed when MtbRho has been pre-incubated with 400 or 500 µM bicyclomycin (400,500; lanes 3, 4). EcRho also caused termination (Ec; lane 5), at a different region of transcript. The terminated transcripts are indicated with dashed lines.

Since all the RNA-binding and ATPase motifs in EcRho and MtbRho are similar, the pTrc99C-*Mtbrho* construct was used to complement the *E. coli* AMO14. The AMO14 genome has an inactivated *rho* gene [48] and a functional copy of *E. coli rho* is supplied on a temperature-sensitive plasmid. The strain is viable at non-permissive temperature (42°C) only when a functional *rho* allele is supplied *in trans*. However, the *E. coli* AMO14 competent cells, transformed with pTrc99C-*mtbrho*, showed no growth at non-permissive temperature (Figure 6A) [22]. Since the presence of the N-terminal subdomain makes MtbRho a larger protein (602residues) than EcRho (419residues) (Figure S1B) [21], [43], it seemed plausible that this additional region of MtbRho could be a hindrance to complementation. However, a deletant of the *MtbRho* gene which lacked the 158-amino acid region (residues 87–241) also failed to complement(data not shown). The failure to complement was not due to lack of expression (Figure 6B). Moreover, as has been reported [22], MtbRho terminated transcription by *E. coli* RNAP *in vitro*. Run-off transcription on a template that contained a pTrc promoter was significantly reduced in presence of MtbRho (Figure 6C). Not only did MtbRho show greater termination efficiency compared to equimolar amounts of EcRho, but also terminated at a region upstream to where EcRho could function. The smaller transcripts were products of *bona fide* Rho-dependent termination as pre-incubating MtbRho with bicyclomycin abolished them and restored run-off transcript levels. The results suggest that inability to interact with *E. coli* transcription machinery is not the reason for failure to complement.

## Discussion

MtbRho is an NTPase that can hydrolyse ATP and GTP efficiently in presence of GC-rich RNA. Although a weaker ATPase compared to the well-characterized EcRho, MtbRho is more efficient in utilizing ATP in presence of its natural substrate i.e. mycobacterial RNAs, compared to EcRho. The intrinsically weak ATPase activity of MtbRho suggests that the enzyme could be a poor translocase as ATP hydrolysis is necessary for powering translocation along the nascent transcript towards RNAP [6], [14], [22], [51], [52]. Notably, it is not the first mycobacterial enzyme shown to have slow catalytic rates. The rates of chain elongation of both *M. tuberculosis* RNAP and DNA Polymerase is significantly lower than the corresponding *E. coli* enzymes [39], [53]and the slow rates are considered to be optimized to the slow-growing lifestyle of the bacteria. Thus, the ATPase rate of MtbRho could be an optimized rate evolved so that termination is functionally *in sync* with the slowly transcribing MtbRNAP. A stronger MtbRho ATPase, in contrast, could possibly result in swift and premature termination, which would be catastrophic for gene expression. The ability of MtbRho to hydrolyse ATP in presence of polyA and polyU, RNA substrates that are inaccessible to EcRho, shows that such broad-RNA specificity is a conserved feature of actinobacterial Rho [54], [55]. Such broad specificity for RNAs could be useful in interacting with a larger number of RNA molecules, both for normal, intergenic rho-dependent termination as well as silencing of xenogenic DNA [3], [56]. It may be indicative of the greater importance of Rho-assisted termination in *Mtb* and other actinobacteria, which have fewer intrinsic terminators [41]. The inefficient use of CTP by MtbRho also parallels earlier studies with Rho homologs from *M. luteus* and *Streptomyces lividans*. The latter two enzymes also hydrolyzed CTP very inefficiently [54], [55]. This strategy could be possibly an adaptation to spare CTP for transcription of GC-rich transcriptomes of these actinobacteria.

The conserved amino acid composition of the N-terminal additional region is indicative of its functional importance and it has been hypothesized to have a role in binding to GC-rich RNA [7]; *M. luteus* Rho has been shown to terminate transcription by *E. coli* RNAP at sites where EcRho cannot terminate, and this is considered indicative that the ‘larger’ Rho are more efficient for terminating on GC-rich RNAs [42], [54]. Our biochemical assays directly validate the long-held assumption that the N-terminal subdomain of actinobacterial Rho can indeed interact with its natural RNA substrate [6], [7]. The functional importance of the additional N-terminal fragment in MtbRho is further evident from the inability of the truncated protein, which lacks the subdomain, to complement EcRho. It is notable that while N-229 can stably interact with mycobacterial RNA, it failed to bind efficiently to poly-dC80. In contrast, full-length MtbRho can interact with both the substrates. The primary RNA-binding motifs of Rho are known to that have a preference for C-rich, unstructured RNA [18], [57] and their absence in N-229 could have compromised its ability to interact with poly-dC80. It is also possible that the extended N-terminal subdomain has evolved to bind efficiently to longer RNA molecules, such as the *sdaA* RNA in this study. Thus, it seems that MtbRho, while retaining ability to bind to the canonical, C-rich substrates of Rho, has a gain-of-function whereby the extended N-terminal region can facilitate binding to mycobacterial RNA. Since MtbRho is a weaker ATPase, interacting with long RNA could result in efficient spooling of RNA around itself and facilitate catching up with RNAP to bring about termination in a largely ATP-independent manner [22].

The inability of MtbRho to complement the strain *E.coli* AMO14 could be attributed to several reasons. The N-terminal subdomain of MtbRho can facilitate binding to RNA that is not a natural substrate for EcRho. This, in turn, could lead to spurious unregulated termination by MtbRho in the *E. coli* strain. Alternatively, the lower ATPase activity of MtbRho could result in inefficient termination, especially since rate of transcription elongation by *E.coli* RNAP is considerably faster than that of mycobacterial RNAP [39]. Approximately 50% of *E. coli* genes rely on Rho for termination [2]. Hence, delayed or imprecise termination could lead to run-off transcripts, ectopic expression from genomic islands and cryptic prophages [3], [56], [58].

The formation of a distinct hexameric (∼400 kDa) species by glutaraldehyde crosslinking indicates that MtbRho can exist as a hexamer even in absence of its substrate RNA and ATP. DLS and analytical gel filtration studies confirm that at least a fraction of MtbRho exists as hexamer in solution. This hexameric population could provide a ‘readily available’ termination-proficient MtbRho within *Mtb* cells [22]. This is in contrast to EcRho which forms hexamer only under catalytic conditions [9]. Our results show that the oligomerization status of MtbRho is significantly influenced by its interaction with RNA. Although a fraction of MtbRho always exists as a ∼400 kDa hexamer, it becomes the predominant form in presence of RNA. The availability of intracellular RNA would thus, act as a cue and ‘activate’ MtbRho to its functional hexameric form. This, in turn, could achieve a functional coordination between MtbRNAP and MtbRho to ensure an orchestrated transcription elongation and termination. In this context, it is noteworthy that the hexamers formed by two *Mtb* elongation/termination factors seem to have different functional consequences. A monomer-hexamer equilibrium, similar to that observed for MtbRho, has also been seen for *Mtb* Mfd protein although the trigger that causes a shift towards monomer or hexamer was not yet identified. The monomeric form of *Mtb* Mfd protein is considered to be the functional one, while the hexamer is likely to be a ‘storage state’ [59]. In case of MtbRho, it appears that nascent RNA could be driving the oligomerization of the termination factor to its functional form.

## Methods

### Bacterial strains, plasmids, construction of clones and chemicals

*E. coli* strains DH10B and BL21(DE3) were used for cloning and overexpression of proteins respectively. The temperature-sensitive *E. coli* AMO14 strain was used for complementation studies [49](Table S1). The *rho* gene was PCR-amplified using *M. tuberculosis* H37Ra genomic DNA as template, primers MtbRhoF and MtbRhoR (Table S1) and Pfu DNA polymerase. The PCR product was first cloned into the EcoRV site of pACYC184 and subsequently mobilized into the NcoI-BamHI sites of pET11d by digesting with RcaI and BglII. For complementation studies, *rho* was similarly cloned into the NcoI-BamHI sites of pTrc99C vector(Table S1). A deletant of MtbRho that lacks the subdomain (ΔNTSrho) was cloned by megaprimer mutagenesis. Primers (ΔNTSXhoF and ΔNTSXhoR) were used to introduce XhoI sites at positions 261 and 693 respectively of the *rho* coding sequence in pTrc99C clone. Digestion with XhoI and ligation resulted in an in-frame deletant that lacked residues 87–231. N–229, the N-terminal subdomain of MtbRho, was amplified using primers NTSfwd and NTSrev and cloned into the NdeI and XhoI sites of pET20b. Competent cells of *E. coli* AMO14 strain were transformed with pTrc99C (vector) or pTrc99C-rho DNA. Cultures were grown at permissive (30°C) and non-permissive (42°C and 37°C) temperatures. All restriction enzymes and DNA modifying agents were from New England Biolabs. DNA ligase was obtained from Roche Applied Science.

### Expression and purification of proteins, RNA and polydC-80

The recombinant MtbRho was overexpressed from the pET11d-rho clone in BL21(DE3) cells. Cultures were grown till OD_600_ 0.6, induced with 0.3 mM IPTG for 3 hrs at 37°C and harvested. Cells were resuspended in buffer (20 mM Tris-HCl pH 8, 200 mM KCl, 20 mM EDTA, 5 mM 2-mercaptoethanol, 5% glycerol), lysed by sonication and centrifuged at 100000 g for 2 hrs at 4°C. This was followed by 0.5% polyethyleneimine (PEI)-based precipitation and then 0–40% ammonium sulphate fractionation. The pellet was resuspended in IX TGED buffer (10 mM Tris-HCl pH 7.6, 5% glycerol, 0.1 mM EDTA and 0.1 mM DTT) containing 150 mM KCl, dialysed against the same buffer to remove the excess salt and then loaded onto a pre-equilibrated Hitrap Heparin-sepharose column. Fractions were eluted in a salt range from 150 to 1000 mM KCl. The fractions containing MtbRho were pooled, dialyzed with 1× TGED+150 mM KCl and applied onto a Hitrap SP-sepharose column. The purity of the protein was assessed by electrophoresing on 8% SDS-PAGE, silver staining and mass spectrometry. Fractions which contained purified MtbRho were pooled and dialyzed overnight in storage buffer (1× TGED+100 mM K-glutamate+50%glycerol). EcRho and N-229, both with a C-terminal hexahistidine tag, were expressed in BL21(DE3) cells. The respective lysate was passed through Ni-NTA column (Qiagen), eluted with 500 mM imidazole and further purified to homogeneity using Hitrap-Heparin column. Concentration of the proteins was estimated by Bradford method. The Ni-NTA beads were from Sigma, all columns and materials were form Amersham-GE Healthcare. Mycobacterial RNA was purified as described in [60] and quality assessed by gel electrophoresis and absorbance ratio (260/280 nm). For generation of *sdaA* RNA, a 330 bp region(genomic coordinates: c76257–c75907) downstream of *Mtb sdaA* gene(Rv0069c) was PCR-amplified using forward primer, that contains T7 RNAP promoter sequence (*sdaA*RNAfwd) and *sdaA*RNArev. 1.5 µg of this template was used to transcribe *sdaA* RNA *in vitro* in presence of 0.5 mM NTP(final) and 20 µCi α-^32^P-UTP (Jonaki, BRIT) and T7 RNAP (Fermentas). Unlabeled *sdaA* RNA was synthesized similarly with only 4 mM ‘cold’ NTPs as precursors. Following DNaseI treatment, the product was purified by phenol-chloroform extraction, alcohol precipitation and passing through Sephadex G-50 minicolumn, and its homogeneity was assessed on denaturing-PAGE. Poly-dC80 oligonucleotide was end-labeled with 15 µCi γ-^32^P-ATP (Jonaki, BRIT) by Polynucleotide Kinase(NEB) as per standard protocol and eluted through Sephadex G-25 minicolumn.

### NTPase activity

The hydrolysis of ATP and other NTPs in presence of different RNA substrates was assessed either by thin-layer chromatography (TLC) or by a Malachite Green-based colorimetric assay. Assays were carried out in T-Buffer (Tris HCl pH8 50 mM, MgOAc 3 mM, K-Glutamate 100 mM, DTT 0.1 mM, EDTA 0.1 mM, BSA 0.1 mg/ml, Glycerol 5%) with 100 nM MtbRho and the appropriate RNA in 10 µl reaction volume. Qualitative assays were carried out for 30 min in presence of 5,10 and 20 ng of polyC, polyA, polyU, 1 and 2 µg *M. smegmatis* total RNA. To test the effect of bicyclomycin, the MtbRho was pre-incubated with 500 µM bicyclomycin at 37°C for 10 min. For quantitative assays, 40 ng polyC, 1 µg *M. smegmatis* total RNA and 300 nM *sdaA* RNA were found to be optimal. Protein concentrations used were with respect to hexameric form of proteins. Reaction was initiated by addition of 1 mM of the ATP/NTP. 100 nCi of γ^32^P-ATP (NEN) was used as tracer in radiometric assay to detect formation of inorganic phosphate (Pi), while α^32^P-ATP(NEN) was used to observe if any AMP- or PPi-generating activity was present. The reactions were stopped by adding chloroform. Following centrifugation, the aqueous phase was used to estimate the release of inorganic phosphate(Pi) by resolving on Polyethyleneimine TLC sheets using LiCl (1.2 M) and EDTA(0.1 mM) as mobile phase and visualized with the Typhoon 9200 phosphorimager. For estimating the hydrolysis of the various NTPs in non-radioactive quantitative assays, a plate-based colorimetric assay [61] was used. Quantitation was carried out by scanning in a microplate ELISA reader at 655 nm. The amount of Pi released was calculated from a calibration curve using known concentrations of KH_2_PO_4_. Reactions were carried till 30–40% of ATP got hydrolysed. Kinetic parameters were obtained by plotting values on a Lineweaver Burk plot. For UV crosslinking, MtbRho was incubated with γ-^32^P-ATP alone, or in presence of unlabeled ATP or CTP at 37°C for 10 min, followed by crosslinking on ice for 30 min using a handheld UV-torch (254 nm). No RNA was added.

### *In vitro* transcription

For transcription by *E. coli* RNAP, template containing Trc promoter was amplified using primers FwdTrc and RhoRevpTrc310, using pTrc99C-rho plasmid as template. Reaction was carried out by incubating 50 nM template with 100 nM *E. coli* RNAP in T-buffer, first on ice and then at 37°C for 10 min each, followed by incubation with 175 nM MtbRho or EcRho for 5 min. Transcription was initiated with 100 µM rNTP and 1 µCi α-^32^P-UTP and continued for 30 min before terminating by addition of phenol-chloroform. Following centrifugation, equal volume of gel-loading dye (95% deionized Formamide, 0.05% Bromophenol Blue and 0.05% Xylene cyanol) was added to the aqueous phase, heated at 90°C for 2 min, chilled and resolved on 6% denaturing PAGE. To test the effect of bicyclomycin, the MtbRho was pre-incubated with 400 or 500 µM bicyclomycin at 37°C for 10 min before addition to RNAP-DNA complex.

### Electrophoretic Mobility Shift assay

For qualititative RNA-binding assays, 100 nM of MtbRho and 100, 200, 400 and 800 nM of N-229 fragment were incubated with 15000 cpm of the ^32^P-labeled *sdaA* RNA or polydC-80 on ice for 10 min. The 10 µl reactions were carried out in T-buffer. The RNA-protein complexes resolved from free RNA by 4 or 6% native PAGE (30:0.6::acrylamide:bisacrylamide). Protein concentrations used were with respect to hexameric form.

### Protease protection assays

1 µg MtbRho was incubated in T-buffer with of *E. coli* tRNA (USB) or *M. smegmatis* SN2 total RNA on ice for 10 min and then at 37°C for 5 min, followed by addition of V8 protease and incubation for different time-points. The reaction was stopped by the addition of inhibitor Tosyl Lysine Chlromethyl Ketone (TLCK, Sigma) and 6× SDS gel loading dye, resolved on 8% SDS-PAGE and visualized with silver staining method.

### Chemical crosslinking

2 µg MtbRho was crosslinked by the addition of glutaraldehyde, to a final concentration of 0.05%. The reactions were carried on for 5 and 10 min at 37°C in T-buffer (using KCl, instead of K-Glutamate) and terminated by addition of 2 M Tris, followed by resolving products on a 3.5-8% gradient SDS-PAGE.

### CD spectroscopy

The CD spectra were recorded from 250–200 nm using a JASCO J–810 spectropolarimeter (Japan Spectroscopic Co Inc. Japan) in a 0.2 mm path-length quartz cuvette. T-buffer(identical to that used in NTPase experiments, but without Glycerol) was used and readings were recorded at room temperature.

### Dynamic Light Scattering(DLS) experiments

DLS analysis were carried out on a Viscotex 802 DLS instrument in 12 µl volume quartz cuvette. T-buffer used for the assay was identical to that used for *in vitro* experiments described above, except that it contained no glycerol. Protein concentration of 300 ng/ul was found to be sufficient for the assay. A 10∶1 molar ratio of tRNA: Rho was maintained. Data was analysed using the software Omnisize 3.0, associated with the DLS machine.

### Analytical gel filtration chromatography

Typically, 100 µg MtbRho was loaded onto Superdex S200 column (bed volume 24 ml, fractionation range 10–600 kDa) alone, or after pre-incubating with tRNA (molar ratio 1∶2) and 1 mM ATP, and 1 ml fractions were collected. After electrophoresing fractions on 8% SDS-PAGE, the eluted protein was assessed by silver staining.

## Funding

V.N. is recipient of J.C. Bose Fellowship from Department of Science and Technology, Government of India. The funders had no role in study design, data collection and analysis, decision to publish, or preparation of the manuscript.

## Supporting Information

Figure S1 **Occurrence and divergence of Rho in bacteria.** (A) Phylogenetic distribution of Rho from several bacterial phyla. (B) Alignment of representative Rho homologues from different bacteria. The 150–200 residue-long subdomain present in the N-terminal half of the actinobacterial homologs is enclosed in red. The alignment was done using the MACAW software. Conserved motifs and residues are shown as boxes and shaded regions.
(TIF)

Figure S2 **Expression and purification of MtbRho.** MtbRho was expressed from pET11d-*rho* clone in BL21(DE3) cells by induction with 0.3 mM IPTG (lane 2). MtbRho purification was carried as described in Methods. The purity of the protein was assessed by silver staining and mass spectrometry.
(TIF)

Figure S3 **Inefficient binding of N-229 to poly-dC80.** N-229 was incubated with the ^32^P-labelled polynucleotide, and the complexes (RC) resolved from free polynucleotide (pC) by 6% native PAGE with 20% glycerol included as stabilizer both in gel and running buffer. No protein (lane 1); 0.6, 1.2 and 1.8 µM of N-229 was used (lanes 2,3,4); presence of excess unlabeled poly-dC80 competed with binding (lane 5).
(TIF)

Figure S4 **Conformational changes of MtbRho upon binding to RNA and ATP.** (A) MtbRho was incubated with *M. smegmatis* RNA and *E. coli* tRNA were used alone, or with ATP and probed with V8 protease. The presence of RNA (lanes 4 to 9; thin arrows) conferred substantial protection against proteolysis, when compared to only MtbRho (lane 3; bold arrows). The addition of ATP along with RNA (lanes 10,11) did not result in any additional or altered protection and ATP alone (lane 12) did not protect MtbRho from protease. (B) Changes in secondary structure were monitored at wavelengths between 200–250 nm in presence of RNA (I) or ATP (II). The molar ellepticity of CD spectra are shown for MtbRho alone (black), in presence of RNA(blue) and in presence of ATP (red).
(TIF)

Table S1 **Strains, plasmids and oligonucleotides used in this study.**
(DOCX)
