# Supplementary figures and images for: Mycobacterium tuberculosis Rho Is an NTPase with Distinct Kinetic Properties and a Novel RNA-Binding Subdomain
Source: PLoS One. 2014 Sep 17;9(9):e107474. doi: 10.1371/journal.pone.0107474 (PMC4167861; doi:10.1371/journal.pone.0107474)

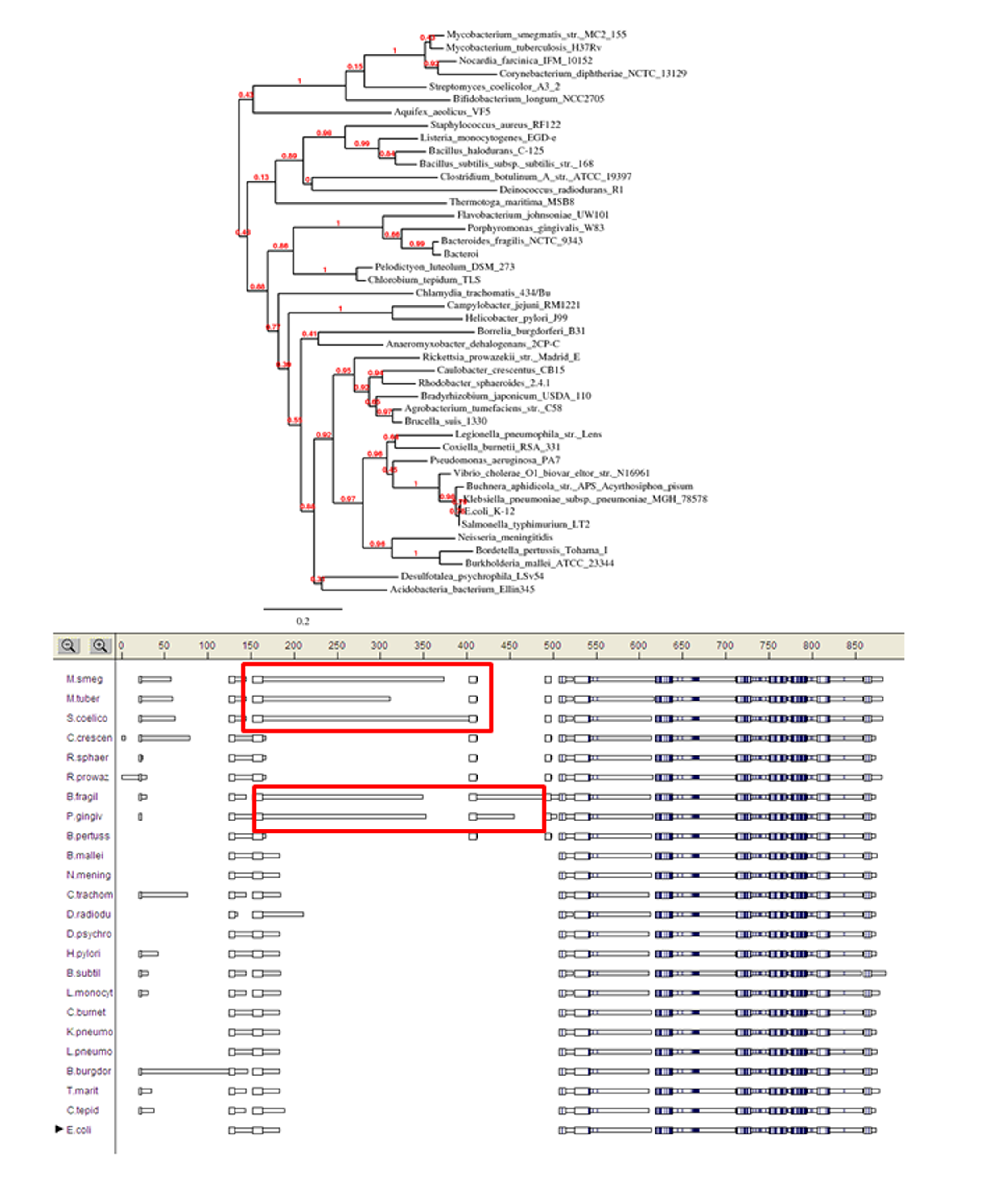

Supplement: Figure S1 — Occurrence and divergence of Rho in bacteria. (A) Phylogenetic distribution of Rho from several bacterial phyla. (B) Alignment of representative Rho homologues from different bacteria. The 150–200 residue-long subdomain present in the N-terminal half of the actinobacterial homologs is enclosed in red. The alignment was done using the MACAW software. Conserved motifs and residues are shown as boxes and shaded regions. (TIF) [file pone.0107474.s001.tif]

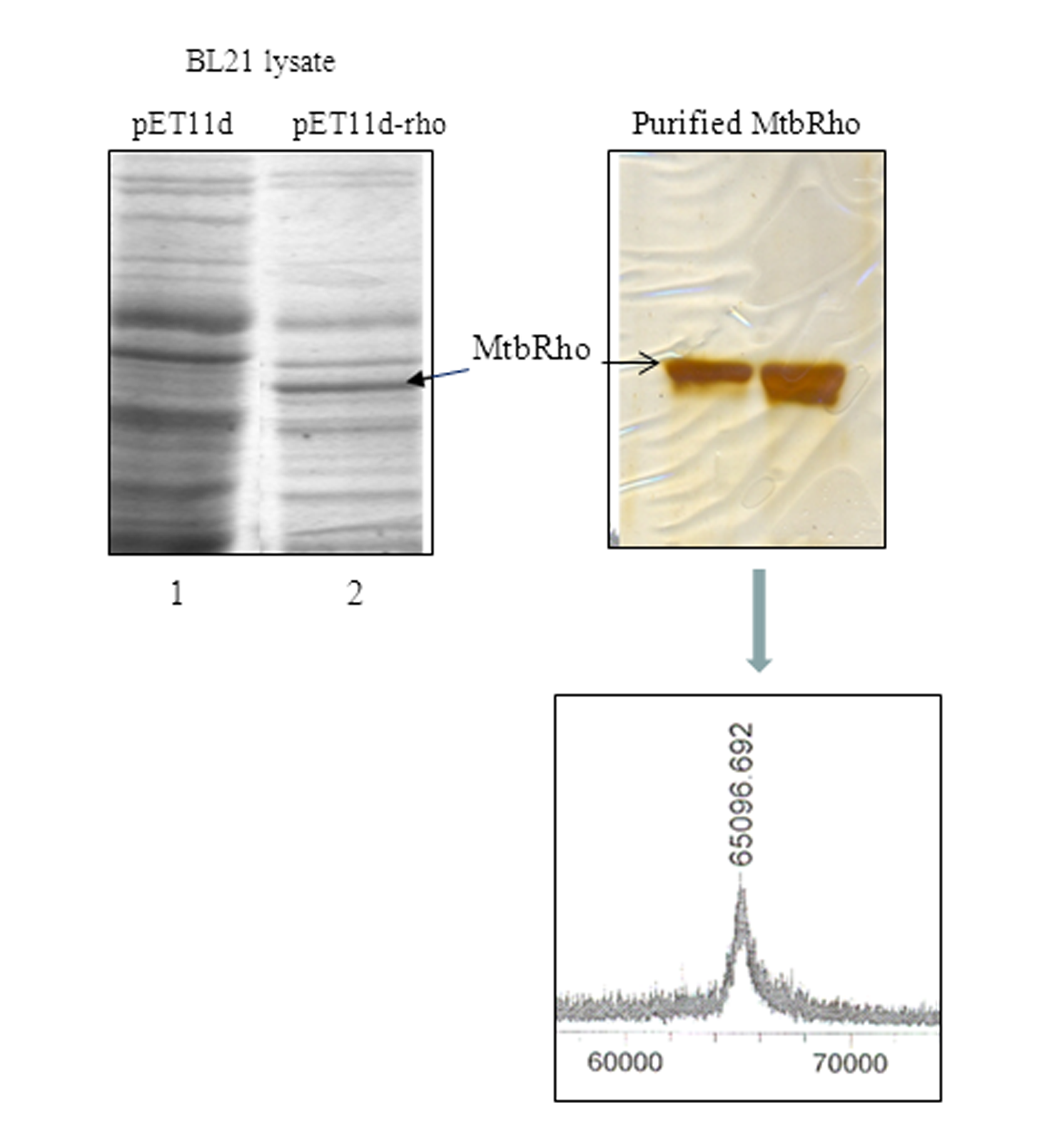

Supplement: Figure S2 — Expression and purification of MtbRho. MtbRho was expressed from pET11d-rho clone in BL21(DE3) cells by induction with 0.3 mM IPTG (lane 2). MtbRho purification was carried as described in Methods. The purity of the protein was assessed by silver staining and mass spectrometry. (TIF) [file pone.0107474.s002.tif]

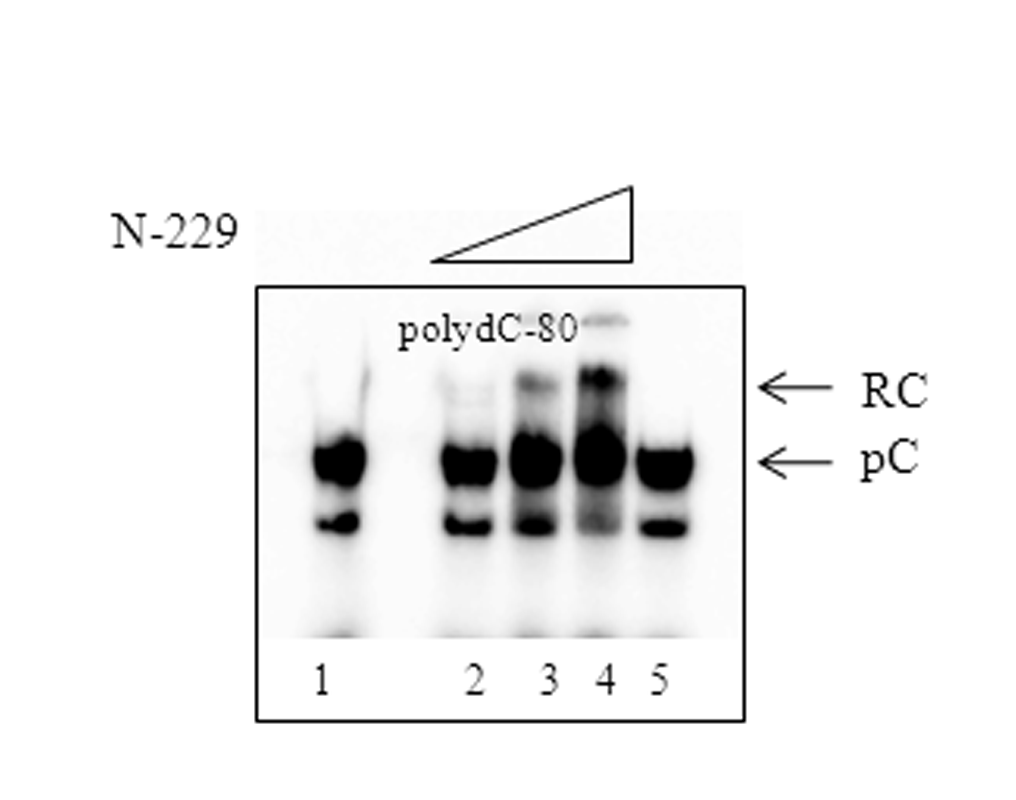

Supplement: Figure S3 — Inefficient binding of N-229 to poly-dC80. N-229 was incubated with the 32P-labelled polynucleotide, and the complexes (RC) resolved from free polynucleotide (pC) by 6% native PAGE with 20% glycerol included as stabilizer both in gel and running buffer. No protein (lane 1); 0.6, 1.2 and 1.8 µM of N-229 was used (lanes 2,3,4); presence of excess unlabeled poly-dC80 competed with binding (lane 5). (TIF) [file pone.0107474.s003.tif]

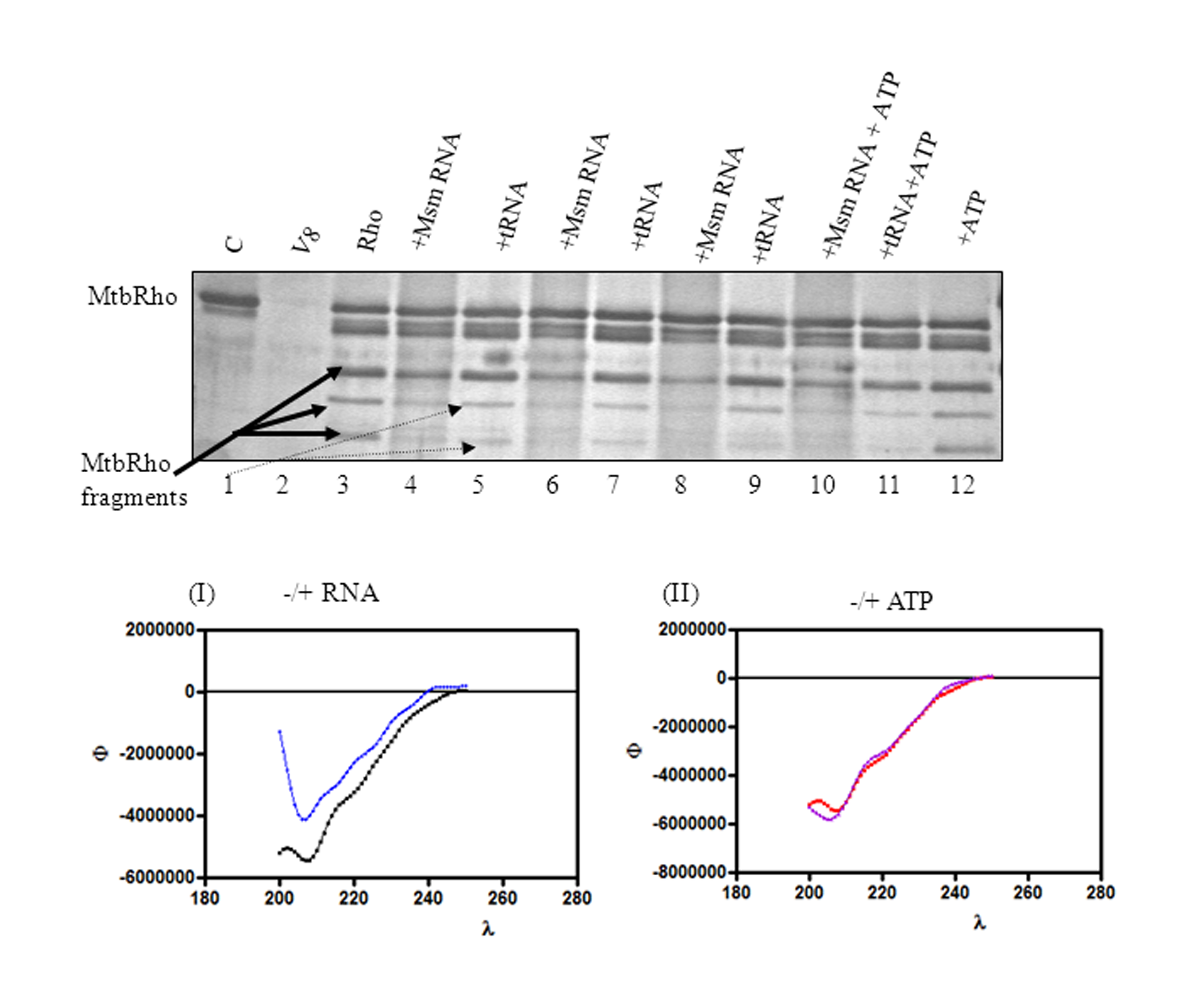

Supplement: Figure S4 — Conformational changes of MtbRho upon binding to RNA and ATP. (A) MtbRho was incubated with M. smegmatis RNA and E. coli tRNA were used alone, or with ATP and probed with V8 protease. The presence of RNA (lanes 4 to 9; thin arrows) conferred substantial protection against proteolysis, when compared to only MtbRho (lane 3; bold arrows). The addition of ATP along with RNA (lanes 10,11) did not result in any additional or altered protection and ATP alone (lane 12) did not protect MtbRho from protease. (B) Changes in secondary structure were monitored at wavelengths between 200–250 nm in presence of RNA (I) or ATP (II). The molar ellepticity of CD spectra are shown for MtbRho alone (black), in presence of RNA(blue) and in presence of ATP (red). (TIF) [file pone.0107474.s004.tif]
