# Supplementary material for: Mycobacterium tuberculosis Rho Is an NTPase with Distinct Kinetic Properties and a Novel RNA-Binding Subdomain
Source: PLoS One. 2014 Sep 17;9(9):e107474. doi: 10.1371/journal.pone.0107474 (PMC4167861; doi:10.1371/journal.pone.0107474)
Supplement: Table S1 — Strains, plasmids and oligonucleotides used in this study. (DOCX) [file pone.0107474.s005.docx]

| **Strains** | Description | Source |
| --- | --- | --- |
| *E. coli* BL21(DE3) | *(hsdS gal (lcIts857 ind1 Sam7 nin5 lacUV5-T7 gene 1)* used for expression and purification | Laboratory stock |
| *E. coli* DH10B | Δ*(mrr-hsd RMS-mcrBC) mcrA recA1*) used for cloning | “ |
| *E. coli* AM014 | Strain with chromosomal *rho* gene inactivated*, rho* supplied in *trans* on plasmid with temperature-sensitive origin of replication | Martinez et al , 1996 |
| **Plasmids** |  |  |
| pET11d-Mtbrho | *M. tuberculosis rho* cloned in NcoI and BamHI sites of pET11d | This work |
| pTrc99C-Mtbrho | *M. tuberculosis rho* cloned in pTrc99C | “ |
| pTrc99C-ΔNTSrho | *M. tuberculosis rho,* with N-terminal subdomain deleted*,* cloned in pTrc99C | ‘’ |
| pET21a-EcRho | *E. coli* rho with C-terminal hexahistidine tag, cloned in pET21a | Epshtein et al, 2010 |
| pET20b-N229 | *M. tuberculosis* rho N-terminal subdomain cloned in NdeI and XhoI sites of pET20b | This work |
| **Oligonucleotides** |  |  |
| MtbRhoF | 5’GGAAATTCATGACCGATACGGAC3’ | This work |
| MtbRhoR | 5’ACACCGAGATCTGTGCGGACATTCCC3’ | “ |
| ΔNTSXhoF | 5’ GACCGGTCGGCTCTCGAGCACGACAAG 3’ | “ |
| ΔNTSXhoR | 5’ GTTGTCCAGGACCTCGAGTATGCC 3’ | “ |
| NTSfwd | 5’ ACTGACATATGACCGATACGGACCTC 3’ | “ |
| NTSrev | 5’ GGACCTCGAGTATGCCGGCTAC | “ |
| sdaARNAfwd | 5’GAAATTAATACGACTCACTATAGGGAGAGTCAACATCGTCGAGTGTTG 3’ | “ |
| Poly-dC80 | 5’ (dC)_80_ 3’ | “ |
| sdaArev | 5’ GTCGCGTGCCACCCAGGAGTG 3’ | “ |
| FwdTrc | 5’cgctcaaggcgcactcccgttc 3’ | “ |
| RhoRevpTrc310 | 5’ GTGGCCGGTGCCTCGGAACTG 3’ | “ |
